# Mitochondrial DNA disease—molecular insights and potential routes to a cure

**DOI:** 10.1016/j.yexcr.2014.03.012

**Published:** 2014-07-01

**Authors:** Oliver Russell, Doug Turnbull

**Affiliations:** Newcastle University Centre for Brain Ageing and Vitality and Wellcome Trust Centre for Mitochondrial Research, Institute for Ageing and Health, The Medical School, Newcastle University, Framlington Place, Newcastle upon Tyne NE2 4HH, UK

**Keywords:** Mitochondrial disease, Treatments, Zinc-finger nucleases, TALENs

## Abstract

Mitochondrial DNA diseases are common neurological conditions caused by mutations in the mitochondrial genome or nuclear genes responsible for its maintenance. Current treatments for these disorders are focussed on the management of the symptoms, rather than the correction of biochemical defects caused by the mutation. This review focuses on the molecular effects of mutations, the symptoms they cause and current work focusing on the development of targeted treatments for mitochondrial DNA disease.

## Highlights

• We discuss several common disease causing mtDNA mutations.
• We highlight recent work linking pathogenicity to deletion size and heteroplasmy.
• We discuss recent advances in the development of targeted mtDNA disease treatments.

## Introduction

Mitochondria are dynamic organelles responsible for producing the majority of ATP, the cellular unit of energy, by a process called oxidative phosphorylation (OXPHOS). This process relies on the transport of electrons across 4 protein complexes in the inner mitochondrial membrane to create an electrochemical gradient. The final OXPHOS complex, ATP synthase, utilises this gradient to drive the formation of ATP and H_2_O [1]. Mutations in mitochondrial DNA (mtDNA), a circular 16.6 kb intronless multi-copy genome found within the mitochondrial matrix, can compromise the production of ATP [2,3]. This genome encodes 13 hydrophobic proteins that are essential subunits of OXPHOS complexes (I,III, IV & V), along with 22 tRNAs and the 2 rRNAs essential for their translation [4].

### Mitochondrial DNA mutations

**Fig. 1:**
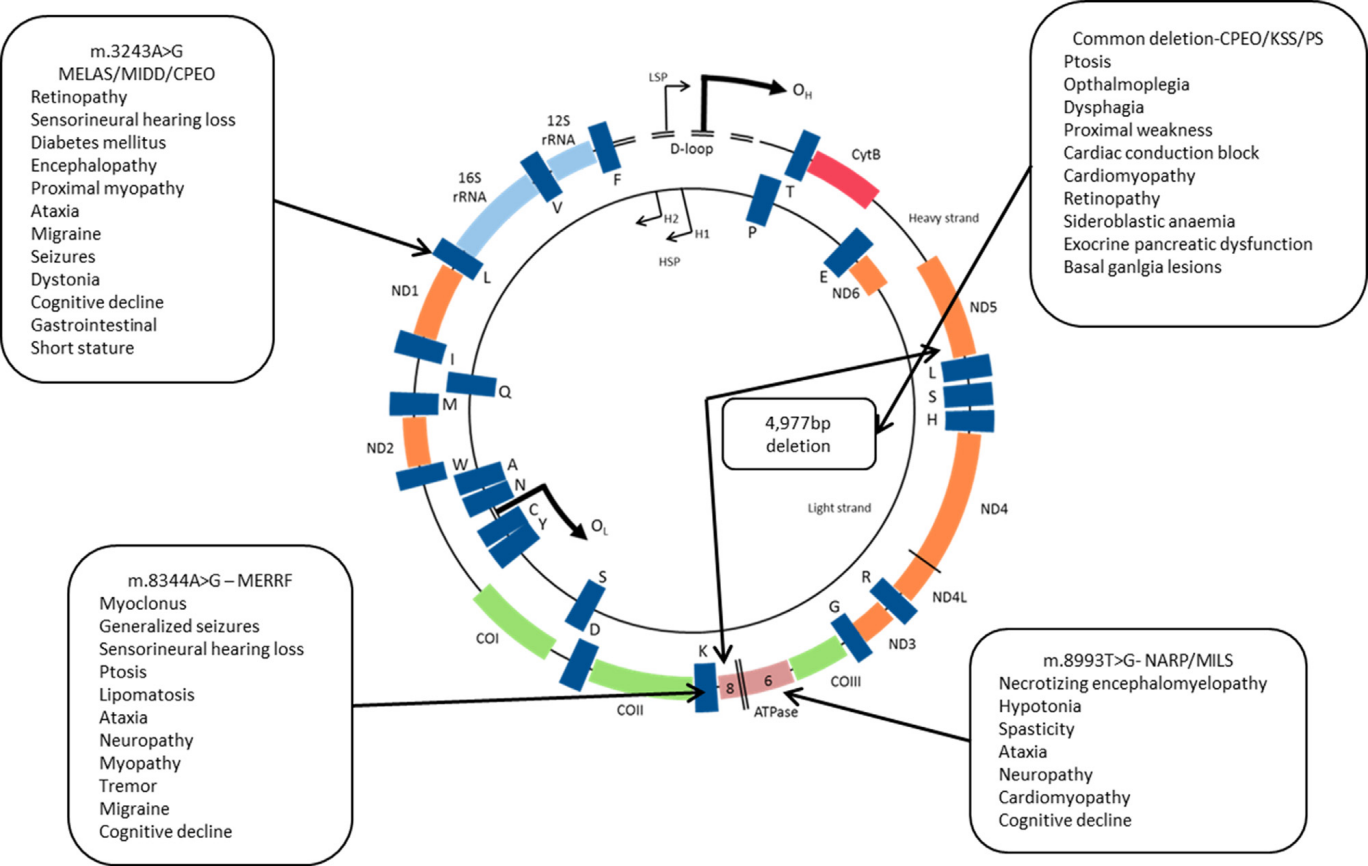
Locations of common disease causing mutations and symptoms they cause. Point mutations are represented by arrows; the “common deletion” is shown in the centre of the genome. Abbreviations: CPEO – chronic progressive external opthalmoplegia, LS – Leigh syndrome, MELAS – mitochondrial encephalopathy, lactic acidosis, stroke-like episodes, MERRF – Myoclonic epilepsy and ragged red fibres, MILS – maternally inherited Leigh syndrome, NARP – neurogenic weakness, ataxia and retinitis pigmentosa, PS – Pearson׳s syndrome.

Mutations of mitochondrial DNA (mtDNA) include point mutations and deletions, the latter of which can arise either from a primary mutation or a mutation in mtDNA maintenance genes (this can also lead to mtDNA depletion) [5,6]. Pathogenic mutations in mtDNA primarily affect the mt-tRNAs and protein encoding regions and a large number of mutations have now been characterised. Although mtDNA has been retained through evolution, nuclear DNA (nDNA) encodes the majority of mitochondrial proteins, including the transcription and replication machinery for mtDNA. As a consequence of this, several mutations of nDNA genes essential for mtDNA maintenance have been described which subsequently cause mtDNA point mutations, multiple deletions or mtDNA depletion [7–10]. The complex interactions between nuclear and mitochondrial genomes and the heterogeneity of mitochondrial disease make the development of treatments very challenging. This mini-review describes the characteristics of several common primary mtDNA mutations (Fig. 1) and diseases they cause. Potential anti-genomic treatments for heteroplasmic mutations and the recent advances in this field are also discussed.

### Heteroplasmy and threshold

The multi-copy nature of the mitochondrial genome leads to complicated genetics. MtDNA mutations can be either heteroplasmic (where both mutated and wild type mtDNA co-exist within the cell) or homoplasmic (only mutated species are present). In the presence of heteroplasmy, for a mutated species to cause a phenotypic effect, the proportion of mutated mtDNA and wild-type mtDNA (*heteroplasmy*) needs to increase to a level where wild-type mtDNA can no longer compensate for the biochemical defect [11]. The threshold of mutated mtDNA required to cause a detectable phenotype depends on the mutation type. Broadly speaking, however, single large-scale mtDNA deletions that remove several genes generally require much lower heteroplasmy (~60%) of mutated mtDNA than tRNA point mutations such as m.3243A>G mt-tRNA^Leu(UUR)^ mutation which decreases the rate of protein translation [12], usually requiring heteroplasmy greater than 80% [11]. Pathogenic homoplasmic mtDNA are less common than heteroplasmic mtDNA mutations. However, three common point mutations, m.11778G>A, m.3460G>A and m.14484T>C which cause Leber׳s Hereditary Optic Neuropathy (LHON) are often homoplasmic [13].

### Maternal inheritance and genetic bottleneck

MtDNA is strictly maternally transmitted; thus women with homoplasmic mtDNA mutations transmit the mutation to their offspring. The situation with heteroplasmic mtDNA mutations is significantly more complicated due to the genetic bottleneck early in development. This bottleneck ensures that some mutations, such as single large scale deletions, are rarely transmitted to offspring, but for those that are transmitted, there is a wide variation in the level of heteroplasmy in the offspring [14].

## MtDNA point mutations

Pathogenic mtDNA point mutations have been described in mt-tRNAs, mt-rRNAs and protein encoding genes, causing a wide range of diseases. The presence of a mutation in mtDNA may not necessarily lead to the same disease in affected individuals, generating a wide clinical spectrum which often makes it hard to diagnose the pathogenic cause of the disease without genetic testing.

### m.3243A>G

First described by Goto et al. in 1990, this mutation in mt-tRNA^Leu(UUR)^ is responsible for 80% of MELAS (mitochondrial encephalomyopathy with lactic acidosis and stroke like episodes) cases [15]. The mutation decreases the rate of protein synthesis, thought to be caused by the prevention of 5-taurinomethyluridine modifications of the uridine wobble-position in the tRNA [12]. Other work has also indicated that the mutation may also cause a decrease in tRNA^Leu(UUR)^ and its aminoacylated form, and also cause dimerisation of the molecule [16]. The consequence of decreased protein synthesis is a respiratory chain complex defect, which leads to the clinical symptoms.

Although this mutation is best known for its associations with MELAS, in fact relatively few patients with the mutation develop this phenotype. Analysis of the UK MRC mitochondrial disease patient cohort has shown that only 10% of patients with the mutation develop MELAS, whereas 30% have maternally inherited diabetes and deafness (MIDD) [17]. The study also showed that patients can also develop a mixture of phenotypes such as MELAS/MIDD syndrome. Interestingly 28% of patients in the cohort had a mixture of symptoms not consistent with normally associated clinical phenotypes. On the other end of the spectrum, 9% of m.3243A>G carriers had no clinical features [17]. This study highlighted the phenotypic heterogeneity associated with just one mtDNA point mutation, elegantly showing the difficulty of clinical diagnosis.

### m.8344A>G

This mutation, found in mitochondrial mt-tRNA^Lys^, was first described in 1990 and is the cause of 80% of cases of MERRF (myoclonic epilepsy with ragged red fibres) [18]. Similar to the m.3243A>G, this mtDNA mutation affects taurine modification of the uridine-wobble position which causes lysine misincorporation and a decreased protein synthesis rate [19,20].

Although the biochemical effects of this mutation should be similar to m.3242A>G, both mutations lead to OXPHOS deficits; the phenotypic consequences of the defect are generally different, although some patients with m.8344A>G have shown MIDD like features [21]. MERRF patients exhibit myoclonus, generalised epilepsy and ataxia along with ragged red fibres in muscle tissues. They can also develop hearing loss, cognitive decline, peripheral neuropathy, optic atrophy and exercise intolerance. Interestingly the mutation has also been shown to reach near homoplasmic levels in adipose tissue around the neck and upper back where it leads to benign growths in the form of lipomas [22].

### m.8993T>G

The pathogenic effect of this mutation is caused by a Leu 156 Arg amino acid substitution in ATP synthase subunit 6, which is thought to impair the synthesis of ATP. The close proximity of the mutation to Glu 58, an amino acid which is essential for the transfer of protons from the intermembrane space through the complex, was thought to affect the protonation and deprotonation of the residue, inhibiting proton transfer [23]. However, more recently it has been shown that this mutation may in fact alter the ability of *c* ring subunits to rotate [24]. Interestingly, this mutation also creates a unique endonuclease restriction site in affected genomes, something that has been investigated in cell models as a potential therapy for these disorders (see below). It should also be noted that an m.8993T>C mutation can also occur and that this is typically associated with a milder phenotype [25].

First described in relation to NARP (neuropathy, ataxia, retinitis pigmentosa), this mutation has more recently been reported in cases of MILS (maternally inherited Leigh׳s syndrome), a disease that occurs in early life causing respiratory abnormalities, dystonia, optic atrophy and is usually fatal [26]. It is thought that the level of heteroplasmy plays a major role in the phenotypic presentation [27,28]. If a patient has between 90% and 95% heteroplasmy, they generally show NARP symptoms; in cases with higher heteroplasmy it is likely that the patient will have MILS phenotype [29].

## Large scale, single mtDNA deletions

Large-scale deletions of mtDNA are thought to occur during repair and/or replication of the genome [30]. Single large-scale mtDNA deletions are typically sporadic, occurring in the germline, and are responsible for some common mitochondrial diseases, which are discussed below. Although there is heterogeneity of single deletion size, studies have shown that a number of patients have a ~5 kb deletion spanning ATPase 8 to ND5 – the so called “common deletion” nt.8467_13446del4977 [31]. Recent work has linked the size of the deletion and its heteroplasmy to disease severity [32]. This has provided a tool to enable clinicians to predict the progression of patients with single deletions, potentially leading to better management of the diseases [32].

The clinical features associated with single large-scale mtDNA deletion disease are varied, similar to the point mutations described previously. The phenotypes include Pearson׳s syndrome which is an early onset disease associated with sideroblastic anaemia and exocrine pancreatic dysfunction; Kearns Sayre Syndrome which is a multisystem disease occurring in childhood and adolescence; and chronic progressive opthalmoplegia which is a later onset disease with predominantly eye muscle involvement. It is important to recognise that these symptoms represent a spectrum of disease and the majority of patients have symptoms between these defined syndromes. The recent observation showing that both the mtDNA deletion size and heteroplasmy level have a significant role in defining the disease phenotype and clinical progression highlights the important link with the biochemical defect.

## Treatments for mtDNA disease

**Fig. 2:**
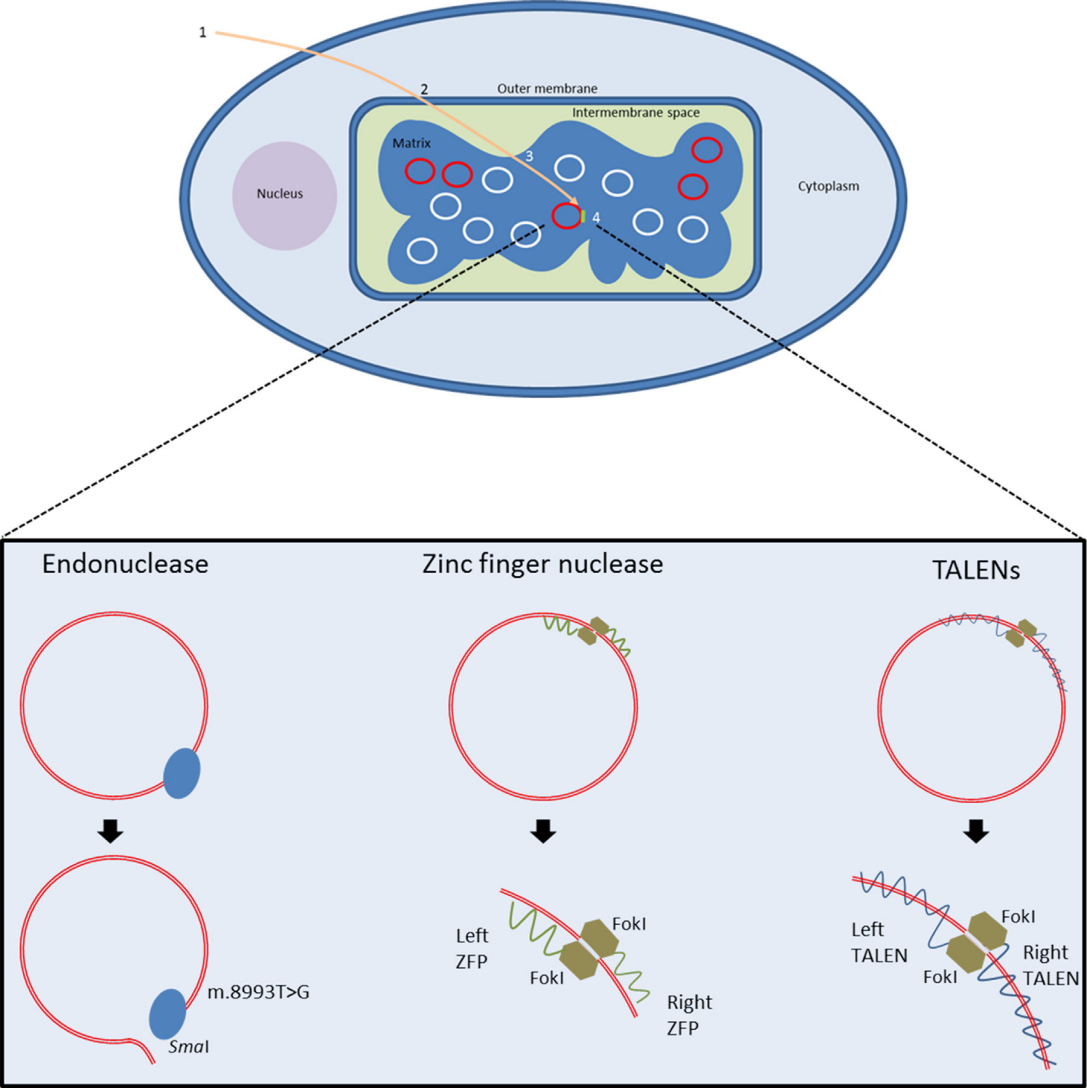
Targeted treatments for mtDNA diseases. A schematic showing the steps required for treatments to access mtDNA. 1. Treatments must be delivered to cell, either as a protein or gene. 2. Selective targeting through mitochondrial membranes. 3. Accumulation in the matrix. 4. Selective targeting of mutated genomes. The binding of three therapies to mtDNA is also shown. *Sma*I endonuclease recognises the m.8993T>G mutation and introduces a double strand break; two ZFNs bind separate regions of mtDNA, one to a mutated area, the other to wild type, enabling their FokI nucleases to interact and cleave mtDNA; TALENs behave in a similar way to ZFPs, requiring two TALENs to bind mtDNA. However, the protein which recognises the mtDNA sequence is longer and wraps around mtDNA.

Although the pace of mitochondrial research has increased since the first description of pathogenic mtDNA mutations in the late 1980s, enabling the identification of hundreds of mtDNA mutations, effective treatments have not been developed yet. Treatment guidelines are generally developed around the supportive management of the diseases rather than their correction, for further information see: http://www.newcastle-mitochondria.com/public-patient/patient-care-guidelines/. This is partly due to the difficulty of delivering treatments to mitochondria; however, as our understanding of mitochondrial transport processes has increased, so has our ability to target molecules to the matrix. In order to treat mtDNA disease, mutated genomes must be prevented from having their pathogenic effects, either by targeted degradation or prevention of replication [33]. Either of these paradigms will shift the balance of heteroplasmy, increasing the amount of wild type mtDNA and correcting the biochemical deficiency. Recent advances in delivering anti-genomic treatments to the mitochondrial matrix of diseased cell lines have been made, all of which have potential to cure mtDNA disease in affected individuals (Fig. 2).

Early efforts to alter heteroplasmy used mitochondrially targeted PNA (peptide nucleic acid) oligomers, DNA with a backbone of amino glycine residues rather than phosphate ribose groups, to specifically bind mutated mtDNA and prevent its replication. Although this work showed promise in vitro, cell line work was less successful due to issues with PNA solubility and delivery [33,34]. The first such therapy to be successfully explored in cell lines was matrix delivery of *Sma*I endonuclease by the addition of mitochondria targeting peptide presequences, small peptides at the N-terminal which cause nuclear encoded mitochondrial proteins to be delivered to the organelle. As mentioned previously the m.8993T>G mutation creates a unique restriction site which is cleaved by *Sma*I enzymes, inducing a double strand DNA break [35]. Delivery of this enzyme to mutant cell lines resulted in a complete depletion of mutated genomes, improving the biochemical defect.

A further advance was the use of Cys_2_–His_2_ zinc finger peptides (ZFPs)— small protein motifs characterised by finger like protrusions that enable the specific binding of small, triplet DNA sequences. These proteins can be designed to recognise any sequence and, with the addition of nucleases to induce double stand breaks in the mutated genome, can be used to remove any disease causing inherited mtDNA mutation. Minczuk et al. have developed ZFPs that are targeted to mitochondria using the COX VII targeting sequence [36,37]. The natural ability of ZFPs to localise to the nucleus meant that the addition of a nuclear exclusion sequence was also required. The authors designed a ZFP that recognised and bound the m.8993T>G NARP/Leigh׳s syndrome mutation. Once bound, the mutated mtDNA was cut by two FokI nucleases that dimerised to induce double strand DNA breaks [36]. This had the effect of causing a stable decline in the heteroplasmy of patient cell lines over a 30-day period [36]. The ability of ZFPs to recognise any sequence makes them very attractive as a potential treatment; however the need for a nuclear exclusion sequence complicates their use.

A further advance in this field was the development of TALEs (Transcription Activator-Like Effectors), a family of proteins which can bind DNA in a sequence specific manner [38,39]. As with ZFPs, TALEs can be modified to include nucleases and targeting sequences. They are more amenable for use in mitochondria as they do not have an innate ability to localise to the nucleus.

Bacman et al. engineered TALEs with FokI nucleases to create TALENs (TALE nucleases) with mitochondrial targeting sequences to bind and cleave the 4977 bp “common deletion” breakpoint (m.8483–13459) in patient derived cell lines [40]. To cut DNA, two TALENs with FokI nucleases were created: one TALEN was complementary to the deletion breakpoint, the other to a region of wild-type mtDNA downstream of the breakpoint. This enabled the FokI nucleases to be in close proximity to dimerise and be cut when bound to mutated mtDNA. Wild-type molecules were not cut as the breakpoint was not present; therefore only one TALEN bound which was not sufficient cut the DNA as only one FokI was present. The authors showed a decrease in heteroplasmy from 70% to 30% in cell lines which contained both TALENs, without affecting the overall copy number of the cells [40].

Although all of these techniques have shown excellent efficacy, the delivery of the recombinant protein is an issue. However, in mitochondrial disorders such as LHON, which primarily affects the eye, transfection may be possible by viral delivery. While the above approaches show that it is possible to remove mutated mtDNA in a targeted manner, the approaches are limited by the lack of efficient delivery mechanisms— something that must be solved in order to deliver these therapies to all affected tissues, in particular to neurons and cardiac muscle.

## Conclusion

The discovery and investigation of pathogenic mtDNA mutations over last 30 years has highlighted the complex relationship between mutation and disease. This heterogeneity makes mitochondrial diseases one of the most challenging neurometabolic diseases to study. The unique biology of mitochondria also makes the development of therapeutics very difficult, and as these two factors combine, it becomes clear why there have been few significant advances in the treatment of these disorders. However, recent advances in the development of anti-genomic therapies have shown that it is possible to directly target the cause of mtDNA disease – mutations in mtDNA. These developments, along with recent advances in reproductive options to women with mtDNA mutations (HFEA review, 2013 – http://www.hfea.gov.uk/6372.html), should offer hope to the patients affected by these disorders that with the right delivery mechanisms, we may one day be able to cure these diseases.
